# Complete genome sequences of four *Phikmvirus* bacteriophages from Kenyan sewage lytic against multidrug-resistant *Pseudomonas aeruginosa*

**DOI:** 10.1128/mra.00795-25

**Published:** 2025-10-10

**Authors:** Meshack Tweya Omwega, Moses Gachoya, Martin Georges, Collins Kigen, James Wachira, Vanessa Natasha, Mikeljon P. Nikolich, Kevin Kamanyi, Samuel Nyamweya, Erick Odoyo, Lillian Musila

**Affiliations:** 1 Kenya Medical Research Institute, Kericho, Kenya; 2 Department of Applied Health Sciences, Kisii University 217802, Kisii, Kenya; 3 Microbiology Hub Kericho, Walter Reed Army Institute of Research – Africa, Kericho, Kenya; 4 Wound Infections Department, Bacterial Diseases Branch, Center for Infectious Diseases Research, Walter Reed Army Institute of Research 8394, Silver Spring, Maryland, USA; 5 Department of Medical Biochemistry & Parasitology, Kisii University 217802, Kisii, Kenya; 6 Department of Pathology, Egerton University 107852, Nakuru, Kenya; Loyola University Chicago, Chicago, Illinois, USA

**Keywords:** bacteriophages, bacteriophage therapy, *Pseudomonas aeruginosa*, *Phikmvirus*, multi-drug resistance, genomic characterization, Kenya

## Abstract

We describe four *Phikmvirus Pseudomonas* phages isolated from sewage samples in Kenya: vB_PaePA01phi1_RS1, vB_PaePA10145Phi1_RS1, vB_PaePA8132phi1_PS3, and vB_PaePA10145phi1_HR2. Their genomes range from 43,723 to 45,485 bp with 62.24%–62.37% GC content and 65–68 coding sequences.

## ANNOUNCEMENT

Hospital-acquired *Pseudomonas aeruginosa* exhibits high resistance to antibiotics, which can cause severe mortality rates and disease severity in hospitalized patients (1, 2). In the context of antibiotic resistance, bacteriophages are promising as potential alternatives to conventional antibiotics (2). We report the complete genome sequences of four lytic phages against *P. aeruginosa*.

Phages vB_PaePA01phi1_RS1, vB_PaePA10145phi1_RS1, vB_PaePA8132phi1_PS3, and vB_PaePA10145phi1_HR2 were isolated in 2022 from four distinct 500 mL grab sewage samples collected on different days from informal settlements in Nairobi, Kenya. The sewage samples were filtered (0.22 µm) to remove bacteria before enrichment in Tryptic Soy Broth (37°C, 180 rpm, 18 h), with the *P. aeruginosa* strains PA01, MRSN 10145, or MRSN 8132 (Table 1). The enrichment culture was centrifuged and filtered (0.22 µm) to obtain the phages (3) which were then purified through three rounds of sequential single-plaque isolation using the double-layer agar method. High-titer stocks were prepared by infecting host cultures at a Multiplicity of Infection (MOI) of ~0.01 until complete lysis (4–6 h), followed by centrifugation and filtration (4). Prior to phage DNA extraction, the bacterial host DNA and RNA were removed with DNase I and RNase A, respectively. The QIAmp Mini Kit (Qiagen, Germantown, MD, USA) was used following the manufacturer’s instructions. DNA purity and quantity were assessed using a Nanodrop One Spectrophotometer and a Qubit fluorometer (Thermo Fisher Scientific Inc., Waltham, MA, USA), respectively. Paired-end sequencing libraries were prepared using the Illumina DNA Prep Kit and whole-genome sequencing on the Illumina NextSeq 1000 (Illumina Inc., San Diego, CA, USA) using a Reagent Kit (300 cycles, 2 × 150 bp reads).

**TABLE 1:** Genomic characteristics of *P. aeruginosa* phages

Phage name	Isolation strains	Sample site (GPS coordinates)	Family	Genus	Genome length (bp)	G + C content (%)	No of CDS	Genome coverage (×)	Genome completeness (%)	No of raw reads	Proportion of mapped reads (%)	Termini and packaging mechanism	GenBank accession	SRA accession no.
Pseudomonas phage vB_PaePA01phi1_RS1	PA01	1°14′26.6″S 37°01′01.9″E	*Autographiviridae*	*Phikmvvirus*	45,485	62.24	67	92.4	100	988,724	99.86	DTR (Long)	PV565004	SRR33007766
Pseudomonas phagevB_PaePA10145phi1_RS1	MRSN 10145	1°14′26.6″S 37°01′01.9″E	*Autographiviridae*	*Phikmvvirus*	43,895	62.31	68	543.1	100	871,199	99.86	Headful (pac)	PV565005	SRR33007765
Pseudomonas phagevB_PaePA8132phi1_PS3	MRSN 8132	1°19'16.4"S, 36°53'07.1"E	*Autographiviridae*	*Phikmvvirus*	43,723	62.35	65	464.4	100	1,442,529	99.84	COS (5’)	PV565006	SRR33007764
Pseudomonas phage vB_PaePA10145phi1_HR2	MRSN 10145	1°19'05.3"S 36°48'23.7"E	*Autographiviridae*	*Phikmvvirus*	43,971	62.26	68	1,098.6	100	1,710,741	99.57	Headful (pac)	PV565007	SRR33007763

FastQC v0.11.9 (https://www.bioinformatics.babraham.ac.uk/projects/fastqc/) examined read quality. Raw reads were trimmed using BBduk v38.18 and Dedupe v.38.18 (https://github.com/BioInfoTools/BBMap). Trimmed reads were assembled using SPAdes v4.0.0 (5), and assembly quality assessed by QUAST v5.3.0 (6). The proportion of mapped reads was determined by Bowtie2 v2.5.4 (7). Genomes were annotated with Pharokka v1.7.4 (8), MMseqs2 v13.45111 (9), and Phanotate v1.6.6 (10–12). Phage lifestyle was predicted using PhageLeads (https://phageleads.dk/) (13), PhageTerm v1.0.12 (14) was used to determine termini and packaging mechanisms, and CheckV v1.0.3 (15) was used for genome completeness. Classification based on Mash distance to the top hits was determined against the INPHARED database v1.8 (16). Default parameters were used for all software.

**Fig 1:**
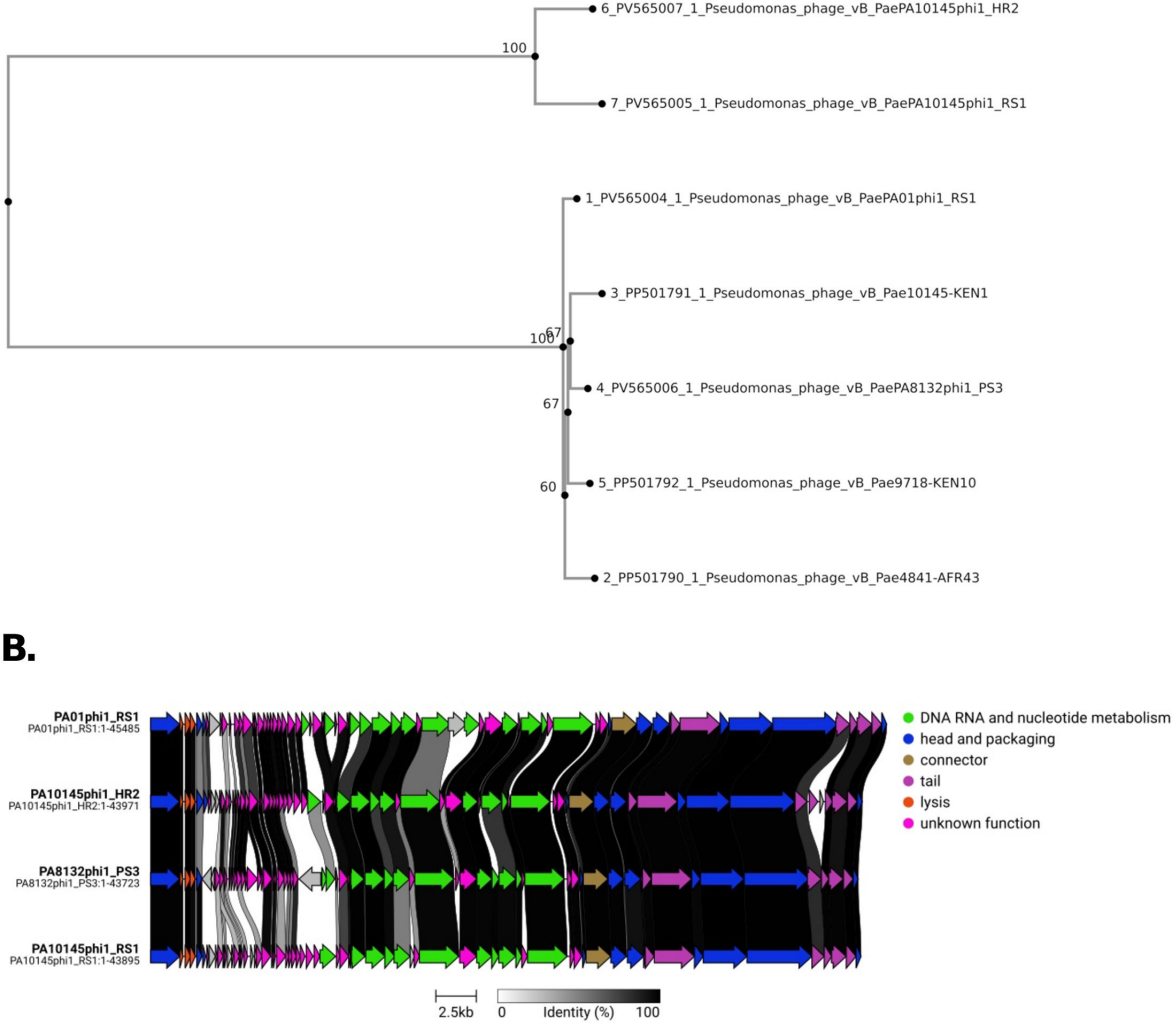
(**A**) Whole-genome phylogenetic tree of seven bacteriophages constructed using the Neighbor-Joining method. The analysis includes the four phages isolated in this study together with three closely related phages previously reported by Peters et al. (17). Bootstrap support values are shown at the major nodes. (**B**) CDS cluster comparison of the four *Phikmvirus* phages generated with Clinker (https://github.com/gamcil/clinker). Greyscale links between genomes indicate amino acid identity, while similarity groups are highlighted with unique colors.

The characteristics of these phages are summarized in Table 1. Comparative genomic analysis (96%–98% similarity NCBI databases) classified the four phages in the genus *Phikmvirus*. This is consistent with recently published phages by Peters et al. (17), which share close taxonomic placement (Fig. 1A). No tRNAs were detected in these phages, typical of this genus (18, 19). Genome comparison using Clinker v0.0.31 (20) shows gene order conservation while also highlighting unique coding sequence variations (Fig. 1B) which may influence infection dynamics (21).

Functional annotation confirmed essential components for virus structure (22, 23) and the lysis cassettes encoding holins, endolysins, and spanins. All four phages lack genes that confer lysogenic lifestyle antibiotic resistance and toxin generation, making them promising candidates for therapeutic applications (24)

## Data Availability

The genome data for PA01phi1_RS1, PA10145phi1_RS1, PA8132phi1_PS3, and PA10145phi1_HR2 are available through BioSample accession numbers, SAMN47761291, SAMN47761292, SAMN47761293, and SAMN47761294, respectively. The GenBank accession numbers are PV565004, PV565005, PV565006, and PV565007, respectively, and the Sequence Read Archive (SRA) accession numbers are SRR33007766, SRR33007765, SRR33007764, and SRR33007763 respectively.
